# Coherence between electromyographic signals of anterior tibialis, soleus, and gastrocnemius during standing balance tasks

**DOI:** 10.3389/fnhum.2023.1042758

**Published:** 2023-04-18

**Authors:** Anuj Ojha, Gordon Alderink, Samhita Rhodes

**Affiliations:** ^1^School of Engineering, Grand Valley State University, Grand Rapids, MI, United States; ^2^Department of Physical Therapy and Athletic Training, Grand Valley State University, Grand Rapids, MI, United States

**Keywords:** EMG, postural stability, wavelet decomposition, intermuscular coherence, tandem stance

## Abstract

**Introduction:**

Knowledge about the mechanics and physiological features of balance for healthy individuals enhances understanding of impairments of balance related to neuropathology secondary to aging, diseases of the central nervous system (CNS), and traumatic brain injury, such as concussion.

**Methods:**

We examined the neural correlations during muscle activation related to quiet standing from the intermuscular coherence in different neural frequency bands. Electromyography (EMG) signals were recorded from six healthy participants (fs = 1,200 Hz for 30 s) from three different muscles bilaterally: anterior tibialis, medial gastrocnemius, and soleus. Data were collected for four different postural stability conditions. In decreasing order of stability these were feet together eyes open, feet together eyes closed, tandem eyes open, and tandem eyes closed. Wavelet decomposition was used to extract the neural frequency bands: gamma, beta, alpha, theta, and delta. Magnitude-squared-coherence (MSC) was computed between different muscle pairs for each of the stability conditions.

**Results and discussion:**

There was greater coherence between muscle pairs in the same leg. Coherence was greater in lower frequency bands. For all frequency bands, the standard deviation of coherence between different muscle pairs was always higher in the less stable positions. Time-frequency coherence spectrograms also showed higher intermuscular coherence for muscle pairs in the same leg and in less stable positions. Our data suggest that coherence between EMG signals may be used as an independent indicator of the neural correlates for stability.

## 1. Introduction

Balancing a large mass over a small base of support is a major challenge for humans in bipedal and unipedal stances. Knowledge about the mechanics and physiological features of balance for healthy individuals enhances understanding impairments of balance related to neuropathology secondary to aging, diseases of the central nervous system (CNS), and traumatic brain injury, such as concussion. Postural control is not only a summation of static reflexes but also a complex skill requiring the cooperation of dynamic sensory-motor processes. Postural sway dynamics of quiet, upright standing contain correlated physiological fluctuations over multiple time scales, with signals that are inherently time-irreversible, non-linear, and non-stationary (Manor et al., 2010). Postural balance includes the coordination of movement strategies to control the center of mass during both self-initiated and remotely activated disturbances of stability (Horak, 2006). Controlling the body positions by the CNS during upright quiet standing can be explained by the types of motor units, synaptic connectivity, motor unit recruitment, and the modulation of proprioceptive information (Elias et al., 2014).

The amplitude and power spectrum of the electromyography (EMG) signal characterizes the muscle fibers’ membrane properties and the timing of motor unit action potentials (MUAPs), which reflect both peripheral and central properties of the neuromuscular system. The approaches to studying relations between surface EMG and properties of the neuromuscular system may allow for the prediction of the effect of various physiological processes on surface EMG and for the identification of underlying physiology. Some research has demonstrated that different recruitment techniques might lead to similar mean frequency patterns (Farina et al., 2002). The degree of synchronization can be determined by cross-correlation analysis between EMG recordings of two muscles when many motor units are synchronized between muscles (Kilner et al., 2002). Coherence as an index for linear synchronization has been used to study cardiovascular mechanisms (Ropella et al., 1988; Faes et al., 2004). Analysis of coherence between EMG signals provides a means of examining characteristics of common neural inputs to co-contracting muscles during voluntary contraction. Using both surface and intramuscular EMG, coherent activity has been reported under various conditions, across a range of frequencies from hand and forearm muscles (Kilner et al., 1999). Amjad et al. (1997) has used several coherence estimates to examine neurogenic coupling between muscles during finger tremor. The CNS oscillatory drives responsible for this coherent activity are typically characterized within different frequency bands. EMG-EMG coherence has been used in many studies to determine oscillatory input to the muscle (Grosse et al., 2002). EMG-EMG coherence are measures commonly used to investigate descending cortical oscillatory drive to a muscle or common oscillatory inputs to different muscles (Héroux and Gandevia, 2013).

Previously, in control participants, rectified EMG used for coherence analysis during quiet standing demonstrated stronger coherence at lower frequencies, and there was a significant increase in coherence between homologous extensors (lateral gastrocnemius, medial gastrocnemius, and soleus) in both frequency bands (0–5 and 10–15 Hz) (Boonstra et al., 2008). However, rectification of EMG disproportionately affects the lower frequency bands. The rectified surface EMG is a better indicator for the segments of the motor unit synchronization at low contraction strengths than the unrectified EMG (Ward et al., 2013). However, some have claimed that the rectified EMG signal undermines the detection of common drive to motor pools suggesting that the use of unrectified EMG signals is preferred for studies on corticomuscular coherence (McClelland et al., 2012). The rectification of EMG loses the information in the lower frequency bands.

Coherence is a frequency domain association measure that is similar to correlation squared and Rosenberg et al. (1989) suggest it is a superior index to look at neural modulation of muscles. However, Rosenberg’s study focused on the neural signals from muscle spindles. Corticomuscular coherence can clearly be detected without rectifying the EMG, and even if rectification enhances coherence in some circumstances, i.e., low force contractions, the fact that it does so inconsistently is a fundamental problem (McClelland et al., 2014). Bilateral coherence between homologous muscles revealed motor unit synchronization between extensor muscles in two frequency bands, and the synchronization was higher in the lower frequency band for the eyes-closed condition (Boonstra et al., 2008). Gibbs et al. (1995) examined cross correlation and synchronization between lower extremities muscles during bipedal stance. However, there is little research investigating the coherence of the lower leg muscles during tandem stance postures.

The purpose of this research was to measure EMG-EMG coherence for most stable to least stable balance stability conditions and using unrectified EMG. The primary objective of this research was to quantify coherence between muscle signals from the lower leg, i.e., anterior tibialis (AT), medial gastrocnemius (MG), and soleus (S) in different frequency bands to examine common neural input characteristics to muscles in healthy individuals. This study may help to provide a better understanding of how the brain modulates muscle activity, a more representative measure of rhythmic activity across muscles, and a useful measure of CNS impairment that affects static and dynamic control of balance, e.g., post-concussion syndrome.

## 2. Materials and methods

### 2.1. Participants

Electromyography data were collected from six healthy participants (three males, three females; 24.1 ± 3.5 years; height: 167.4 ± 5.5 cm; weight: 71.1 ± 5.5 kg). The research protocol was approved by the Human Research Review Committee, Institutional Review Board, Office of Research Compliance and Integrity, Grand Valley State University (IRB number: 18–246-H). Each participant was recruited as per their interest based on strict exclusion criteria and obtaining an informed consent before participation. Exclusion criteria were:

1. No history of neurological diseases such as Cerebral Palsy, Multiple Sclerosis, etc.
2. No major musculoskeletal injuries/impairments of the pelvis and lower extremities.
3. No persistent symptoms of vertigo, lightheadedness, unsteadiness, or history of concussion within the past year.
4. For female participants, pregnancy.

### 2.2. Instrumentation

Electromyography signals from the lower leg: AT, MG, and S were collected at 1,200 Hz using the Motion Lab System (Motion Lab Systems Inc., Baton Rouge, LA, USA). The MA-411 EMG pre-amplifier was used to record data which incorporates both radio frequency interference filters and electrostatic discharge protection circuitry that helps to eliminate motion artifacts and cable noise providing a reliable EMG signal. The electrode amplifier is a double differential amplifier with Common Mode Rejection Ratio >100 dB at 65 Hz, noise <1.2 μV, and input impedance >100,000 MΩ.

Sixteen Vicon-MX cameras (120 Hz) and Nexus motion-capture software, v2.61 (Oxford Metrics, Oxford, UK) were used to track anatomical marker trajectories. Force and EMG data were synchronized with motion capture data. Ground reaction forces were collected at 1,200 Hz, using floor embedded AMTI force platforms (Advanced Mechanical Technology Inc., Watertown, MA, USA). Although ground reaction force and marker trajectory on body segments were captured, only EMG data were analyzed and presented in this manuscript.

### 2.3. Data collection

A modification of the Full Body Plug-in Gait biomechanical model was used to capture position data.^1^ Prior to attaching the surface EMG amplifier/electrode, body height and mass, and several model-required anthropometric measures were taken. The skin overlying the approximate mid-muscle bellies of the right and left AT (RAT and LAT), MG (RMG and LMG), and S (RS and LS) was cleaned and slightly abraded using mini-alcohol swabs. Electrodes were oriented approximately parallel to muscle fiber orientation for each muscle, according to SENIAM (Surface EMG for a Non-Invasive Assessment of Muscles) recommendations (Hermens et al., 2000). Electrodes were secured using hypoallergenic tape and pre wrap athletic tape to minimize soft tissue and cable artifact. Accuracy of electrode placement was insured by performing ankle joint dorsiflexion (for RAT and LAT) and plantarflexion (for RMG, LMG, RS, and LS) movements. During the placement accuracy testing, signal gains were adjusted in the patient unit to provide an adequate amplitude scale and check for signal amplitude clipping.

Data were collected for 30 s with participants standing on force plates under four different conditions: (1) feet together eyes open (FTEO), (2) feet together eyes closed (FTEC), (3) tandem eyes open (TanEO), and (4) tandem eyes closed (TanEC). The tandem standing position was posed with the participant’s dominant foot placed on a rear force plate and non-dominant foot placed on the fore force plate with approximately 13 cm distance between the heel of the leading foot and the toes of the trailing foot. For the feet together condition, the participant stood with both feet on a single force plate with hips and knees extended and with elbows flexed so that the fingertips touched the anterior shoulders. For tandem conditions, the participants assumed the tandem foot position, knees extended, attempting to maintain equal distribution of weight on each force plate, and holding arms in front with fingertips touching the anterior shoulders.

Five trials were performed under each condition. Participants were provided 30–60 s between trials. In the event of the loss of balance, data collection continued if the participant maintained full contact with the force plates and also in the case of the eyes closed condition, kept eyes closed. If, with loss of balance the participant stepped off the force platforms and/or opened their eyes in the eyes closed condition, a new trial was collected.

### 2.4. Data processing

Raw EMG signals were exported into a custom MATLAB program for further processing. Zero-mean of the signals was performed to remove DC bias. The signal was then notch filtered at 60 Hz to remove line noise. Since we used surface EMG electrodes it is possible that signals from the soleus and medial gastrocnemius were subject to cross-talk (Winter, 2009). The MATLAB “xcorr” function was used to test for cross-correlation, i.e., to test for cross-talk between the muscle pairs. Intermuscular coherence between pairs of muscles was examined in different neural frequency bands as shown in Table 1 using wavelet decomposition, and magnitude-squared-coherence (MSC). A time-frequency coherence analysis was also performed between all muscle pairs.

**TABLE 1:** Frequency range for different frequency bands before and after wavelet decomposition.

Frequency band	Frequency range (Hz)	Frequency range after wavelets decomposition (Hz)
Delta	1–4	1.1719–4.1016
Theta	4–8	4.1016–8.2021
Alpha	8–13	8.2031–13.4766
Beta	13–30	14.0625–32.8125
Gamma	30–100	32.8125–103.1250

#### 2.4.1. Wavelet decomposition

The use of wavelets can improve classification accuracy and decreases total computational time (Phinyomark et al., 2011). Wavelet decomposition was performed using order 45 Daubechies wavelet at eight levels. The five frequency bands examined in this study were gamma, beta, alpha, theta, and delta.

#### 2.4.2. Magnitude-squared coherence

Magnitude-squared-coherence is a function of frequency with values between 0 and 1 and is used to analyze non-stationary signals. Coherence of EMG signals is used to examine common neural inputs to the co-contracting muscles. MSC, *C*_*xy*_(*f*), between two signals *x*(*t*) and *y*(*t*) is defined as


$$C_{x\,y}\,\left(f\right)=\frac{{\left|S_{x\,y}\,\left(f\right)\right|}^{2}}{S_{x\,x}\,\left(f\right)\,S_{y\,y}\,\left(f\right)}$$

where *S*_*xy*_(*f*) is the cross-spectrum, and *S*_*xx*_(*f*) and *S*_*yy*_(*f*) are the auto-spectra of *x*(*t*) and *y*(*t*), respectively.

Magnitude-squared-coherence represents one statistical index to measure the similarities in the frequency content of the two signals, and it can possibly help to eliminate Type I error. MSC between pairs of EMG signals was determined to ascertain if there were systematic changes in muscle coherence in each frequency band due to changes in postural stability related to eyes open or closed conditions.

Magnitude-squared-coherence of signal wavelets was calculated for different muscle pairs under FTEO, FTEC, TanEO, and TanEC conditions for all five frequency bands as mentioned in Table 1. The muscle pairs used for coherence analysis were LAT-LMG, LAT-LS, LMG-LS, RAT-RMG, RAT-RS, LAT-RAT, LMG-RMG, and LS-RS. EMG-EMG coherence was estimated using a Hamming window of length 256 for the gamma band, 512 for the beta band, 2,048 for the alpha band, and 4,096 for the theta and delta bands with a 50% overlap between adjacent windows. Window lengths for each band were chosen to match wavelet lengths at each frequency band.

#### 2.4.3. Time-frequency coherence

Time-frequency coherence analysis of EMG signals was also determined for muscle pairs LAT-LMG, LAT-LS, LMG-LS, RAT-RMG, RAT-RS, LAT-RAT, LMG-RMG, and LS-RS under FTEO, FTEC, TanEO, and TanEC conditions. MSC for time-frequency analysis was calculated using the equation: (Lovett and Ropella, 1997)


$$M\,S\,C\,\left[n,k\right]=\frac{{\left|\sum_{l=0}^{L-1}X_{l}\,\left[n,k\right]\,Y_{l}^{*}\,\left[n,k\right]\right|}^{2}}{\sum_{l=0}^{L-1}{\left|X_{l}\,\left[n,k\right]\right|}^{2}\,\sum_{l=0}^{L-1}{\left|Y_{l}\,\left[n,k\right]\right|}^{2}}$$

where *X*_*l*_[*n*,*k*] and *Y*_*l*_[*n*,*k*] are discrete Fourier transforms of single segments *x*[*n*] and *y*[*n*].

Raw EMG signals, after passing through the notch filter, without the wavelet decomposition were used for time-frequency analysis to examine the patterns of coherence distribution for different muscle pairs at different frequencies. Time-frequency coherence between muscle pairs was obtained up to 100 Hz using a window length of 2,400 with 50% overlap.

#### 2.4.4. Statistical analysis

Two sample *t*-test were used for all muscle pairs in all frequency bands. We were interested if there were mean coherence differences between muscle pairs for different frequency bands. The *p*-value was examined at a 5% significance level. Median MSC between muscle pairs was calculated for each trial and mean and standard deviation of obtained median values was estimated and used for descriptive purposes.

## 3. Results

**FIGURE 1:**
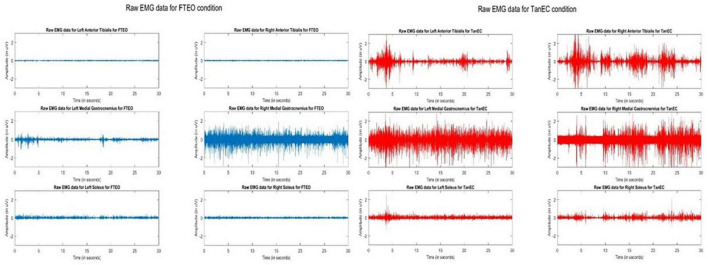
Raw EMG signals of AT, MG, and S from both legs during standing balance under most stable position FTEO (in blue) and least stable position TanEC (in red) from a single trial from one participant. The image shows how the amplitude of EMG signals change from the most to the least stable postural standing positions.

Observation of raw EMG signals during changes in testing conditions suggest that as internal challenges to posture were added, signal density appeared to increase. Figure 1 illustrates raw signals for a representative participant for FTEO and TanEC conditions. In more stable postures it appears that AT muscles were less active, whereas in the most unstable posture the AT, MG, and S were equally active. These observations were consistent for all study participants.

### 3.1. Cross-talk

**FIGURE 2:**
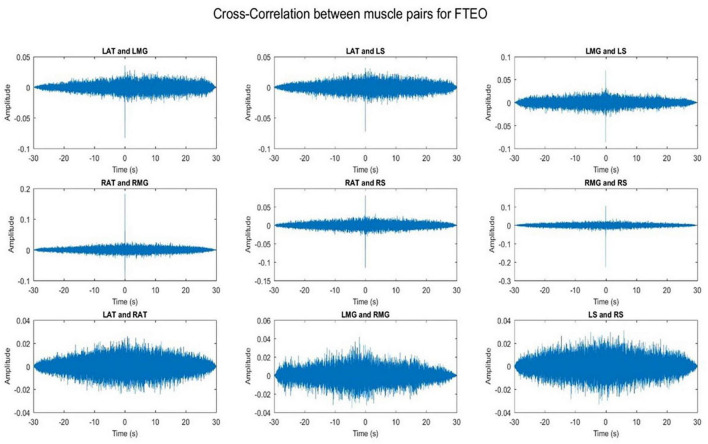
Cross-correlation of different muscle pairs under FTEO condition for a single participant for a single trial. The figure represents cross-correlation between the EMG signals of different muscle pairs under the most stable postural position. It shows negligible cross talk between the muscle pairs.

**FIGURE 3:**
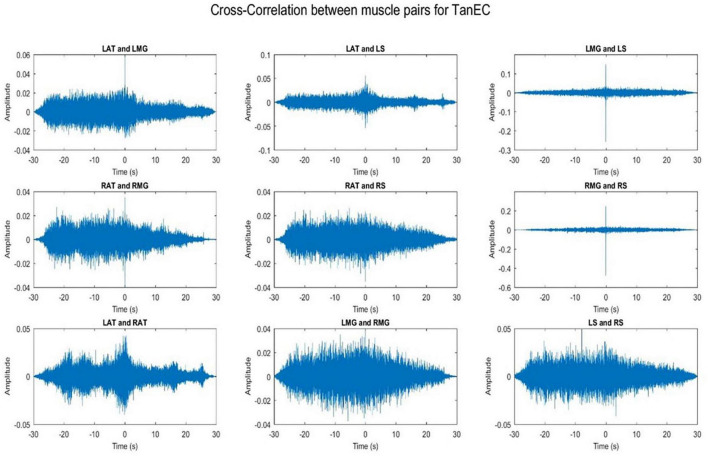
Cross-correlation of different muscle pairs under TanEC condition for a single participant for a single trial. The figure represents cross-correlation between the EMG signals of different muscle pairs under the least stable postural standing position. It shows negligible cross talk between the muscle pairs.

Cross-talk between each muscle pair for one representative participant was conducted. Results indicate that cross-talk was negligible (Figures 2, 3). As a result of this analysis, we believe that our coherence analysis results were not influenced by cross-talk.

### 3.2. Magnitude-squared coherence

**FIGURE 4:**
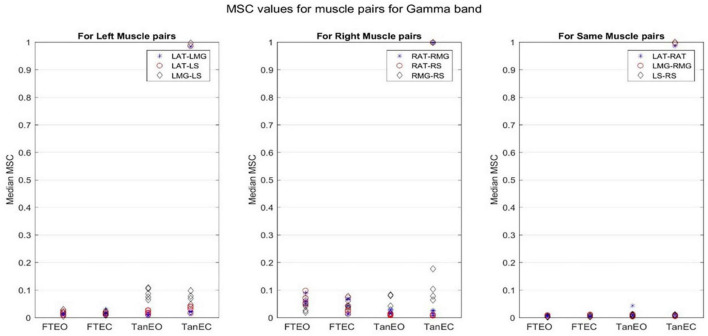
Magnitude-squared-coherence plot for different muscle pairs under different standing conditions for gamma frequency band (32.8125–103. 1250 Hz) for a single participant for all individual trials. The plot shows coherence between different muscle pairs for the most to least stable postural standing positions in the gamma band. Outliers are seen in the data for TanEC conditions.

**FIGURE 5:**
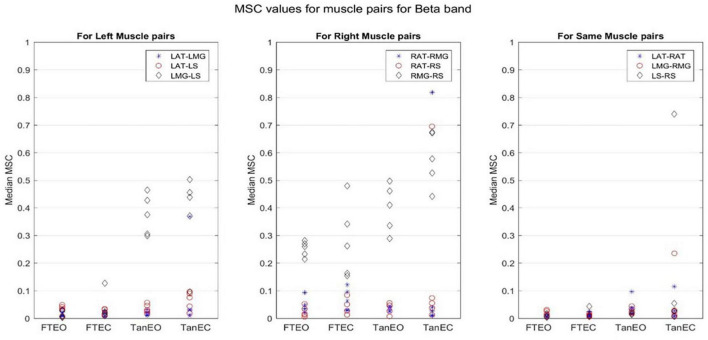
Magnitude-squared-coherence plot for different muscle pairs under different standing conditions for beta frequency band (14.0625–32.8125 Hz) for a single participant for all individual trials. The plot shows coherence between different muscle pairs for the most to least stable postural standing positions in the beta band. It can be seen how coherence for different muscle pairs have increased mainly in the right leg between RMG and RS on moving to lower frequency band.

**FIGURE 6:**
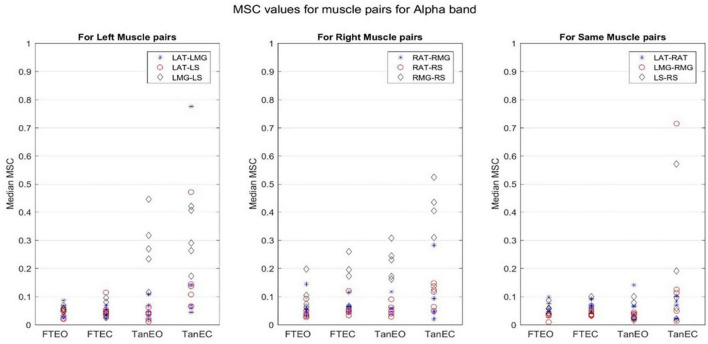
Magnitude-squared-coherence plot for different muscle pairs under different standing conditions for alpha frequency band (8.2031–13.4766 Hz) for a single participant for all individual trials. The plot shows coherence between different muscle pairs for the most to least stable postural standing positions in the alpha band. It can be seen how coherence for different muscle pairs have increased, mainly in the right leg between RMG and RS on moving to lower frequency band.

**FIGURE 7:**
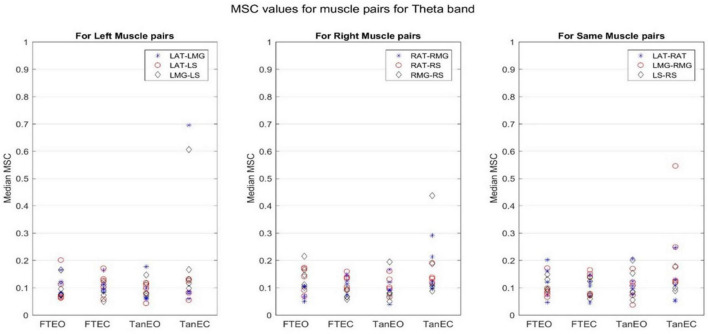
Magnitude-squared-coherence plot for different muscle pairs under different standing conditions for theta frequency band (4.1016–8.2021 Hz) for a single participant for all individual trials. The plot shows coherence between different muscle pairs for the most to least stable postural standing positions in the theta band. It can be seen how coherence for different muscle pairs have increased on moving to lower frequency band.

**FIGURE 8:**
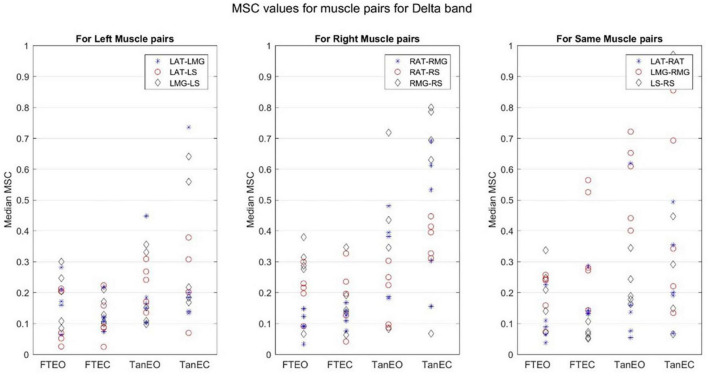
Magnitude-squared-coherence plot for different muscle pairs under different standing conditions for delta frequency band (1.179–4.1016 Hz) for a single participant for all individual trials. The plot shows coherence between different muscle pairs for the most to least stable postural standing positions in the delta band. It can be seen how coherence for different muscle pairs have increased and has the highest coherence value in the lowest frequency band.

Coherence values in each frequency band for a representative participant for each of the four stability conditions are shown in Figures 4–8.

Coherence between muscle pairs trended higher in the least stable postural position TanEC. In general, coherence appeared higher in muscle pairs for the right, i.e., dominant, limb, which was situated over the rear force platform. Furthermore, coherence appeared to decrease slightly moving from lower to higher frequency bands. It also appears that coherence between RMG and RS were greater under all conditions. There was greater coherence between muscle pairs in the delta frequency band. The *p*-value was found to be significant for all muscle pairs at a 5% significance level only in the delta band.

### 3.3. Time-frequency coherence analysis

**FIGURE 9:**
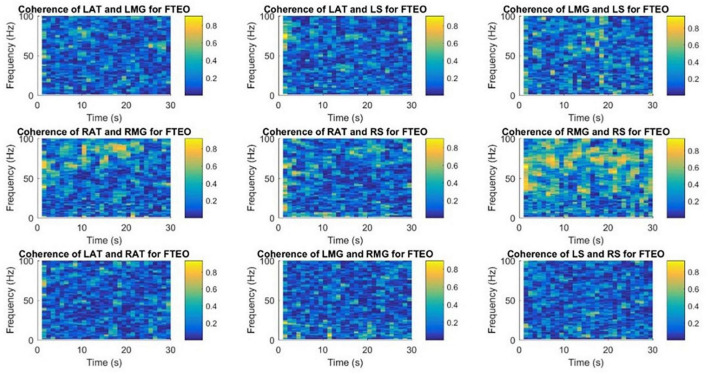
Time-frequency coherence spectrogram of different muscle pairs under FTEO condition for a single participant for a single trial. The figure represents EMG-EMG coherence of different muscle pairs for a particular frequency and time with blue representing the lowest intermuscular coherence and yellow representing higher coherence between muscle pairs under the most stable postural standing position.

**FIGURE 10:**
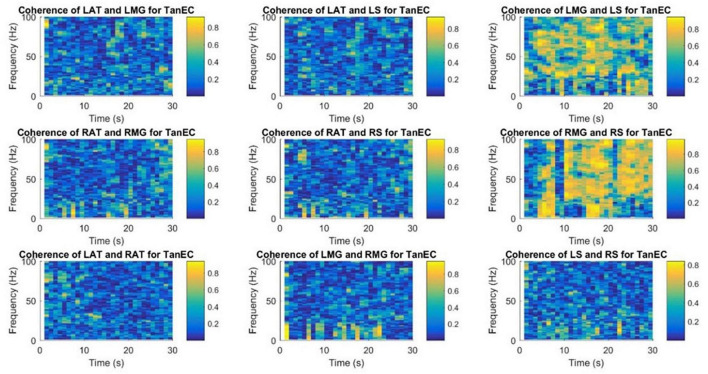
Time-frequency coherence spectrogram of different muscle pairs under TanEC condition for a single participant for a single trial. The figure represents EMG-EMG coherence of different muscle pairs for a particular frequency and time with blue representing the lowest intermuscular coherence and yellow representing higher coherence between muscle pairs under the least stable postural standing position. Note that at all frequencies, the coherence is higher between RMG and RS for TanEC condition.

Figures 9, 10 demonstrate time-frequency coherence spectrogram from 0 to 100 Hz over the 30 s trial for a representative participant for the most stable and the least stable balance condition. Time-frequency analysis indicated stronger muscular coherence, particularly for the two posterior tibial muscles (MG and S), when participants were in the tandem posture. It appears that there was a greater coherence between the RMG and RS, which corroborates the findings of MSC analysis of specific frequency bands.

## 4. Discussion

Our objective was to examine the common neural input characteristics to muscles in individuals without neuropathology by analyzing coherence between muscle signals from important postural control lower leg muscles in different frequency bands in different standing balance tasks. Coherence analysis of surface EMG between different muscles was studied to investigate the common synaptic input to motor neurons. Our results demonstrated a higher coherence between RMG and RS in all frequency bands. Higher overall coherence was found for right- and left-sided muscle pairs under all conditions in lower frequency bands, with the highest coherence for all muscle pairs under all conditions in the delta band. Coherence was higher in all muscle pairs in all frequency bands under the eyes-closed conditions, and generally higher in the tandem stance posture. Results for this study showed a strong coherence in the right leg muscles which may be because all participants were right leg dominant. These data could serve as a control dataset for analysis of individuals with neuropathology involving impaired standing balance.

For all standing balance tasks motor synergies are vital to human motor control. Both peripheral and central properties of the neuromuscular system are reflected through surface EMG. Common synaptic inputs to motor neurons are often ascertained by analyzing coherence between EMG signals. In previous studies, significant EMG-EMG coherence for common inputs was identified at higher frequency bands but no inputs in the delta band were identified by EMG-EMG coherence and the absence of coherence at the delta band might be because of the presence of the power of MUAPs at higher frequencies (Negro et al., 2015; Dideriksen et al., 2018). Our findings support those reported by Gibbs et al. (1995) who used cross-correlation analysis between the EMG signals of soleus and gastrocnemius to determine that the shared action of those muscles around the same joint and the noticeably higher degree of synchronization during balance indicated that these muscles had a common drive.

In the present study, wavelet decomposition was performed to obtain power at different neural frequency bands and intermuscular coherence between different muscle pairs was calculated in each frequency bands. We found that coherence values increased between muscle pairs from higher to lower frequency bands. Intermuscular coherence was greater in an eyes-closed condition and in a tandem foot position rather than standing with feet together. Coherence dispersion increased as we examined data from higher frequency to lower frequency bands and maximum coherence dispersion was observed for the least stable posture TanEC for all frequency bands. The frequency dependent differences in neural drives have been reported by Laine et al. (2015) who studied synergistic motor unit coherence during a knee extension task. They suggested that neurally modulated coherence at low frequencies (6–12 Hz) was associated with afferent feedback while at high frequencies (∼20 Hz) was associated with descending cortical input. Our results therefore suggest that balance may be neurally modulated more at the spinal cord rather than at the cortical level.

Several theories that have attempted to explain the motor performance variability – one of which is the uncontrolled manifold hypothesis (UCM). This hypothesis is associated with how the CNS may attempt to manage the indeterminacy of muscle activations and has been used to describe the coordination strategies of redundant motor systems to perform different motor tasks (Krishnamoorthy et al., 2003a,b; Krishnamoorthy et al., 2004). These groups identified three major functional muscle groups, termed muscle modes, which co-varied their EMG magnitude to provide a stable trajectory of the position of the body’s COP. Results of the series of projects by Krishnamoorthy et al. strongly suggest the involvement of multi-muscle synergies in human postural control. Glass et al. (2022) attempted to characterize affirmative indicators of good postural alignment by combining a modified UCM framework, i.e., angular displacements, and intermuscular coherence. They showed that greater postural angular malalignment was associated with compromises in synergistic control of the vertical posture and increased corticospinal drive to specific muscles. Finally, Torres-Oviedo and Ting (2007) examined how multiple muscle synergies accounted for EMG variability in tasks involving automatic postural responses from different perturbation directions. Their data suggested that trial-to-trial variations in the activation of individual muscles were correlated and represented variations in the amplitude of descending neural commands that activated individual muscle synergies. It is our contention that these studies indicate that combining multiple methodological approaches, i.e., UCM, intermuscular coherence, and EMG matrix factorization, could enrich insights into the control of static and dynamic postures.

In a previous study, stronger coherence was found for lower leg muscles and muscle combinations within the same leg, and intermuscular coherence was significant for agonists and antagonists in the lower leg across a broad range of frequencies (0–40 Hz) (Boonstra et al., 2015). Intermuscular coherence observed at very low frequencies (<5 Hz) in the present study is consistent with other studies on postural control. For example, Danna-Dos-Santos et al. (2014) showed that the soleus and biceps femoris muscle pair had the strongest coherence in the 0–5 Hz frequency range. Both bilateral and unilateral soleus pairings demonstrated coherence in the 0–5 Hz frequency band, which was greater during the postural task (Mochizuki et al., 2006).

In a previous study of bipedal stance, the pooled coherence of anterior, posterior, core, and mixed pairs of muscles and antagonists was significant in the 0–5 Hz frequency band, and in unipedal instance, mixed and anterior groups showed lower coherence values than the posterior, core, and antagonist groups. These EMG-EMG coherence differences during unipedal stance suggest that there are different muscle synergies involved (García-Massó et al., 2016). Coherence increased with greater task difficulty in only agonist-antagonist muscles in the low-frequency band, reflecting subcortical inputs and only in the agonist-agonist group in the high-frequency band, reflecting corticospinal inputs. Therefore, common neural inputs to both agonist-agonist and agonist-antagonist muscles appear to increase with task difficulty but are likely driven by different sources of input to spinal alpha motor neurons (Nandi et al., 2019). During the unipedal instance testing for both young and elderly adults, it has been reported that there is a strong coherence between the medial gastrocnemius and soleus muscles in the delta frequency band (0–5 Hz), and the coherence for other different muscle pairs is stronger in the beta band (15–35 Hz) (Watanabe et al., 2018). These data suggest that the oscillatory activity between the MG and S muscles is strongly involved in control of unipedal stance for elderly people.

During postural control, intermuscular coherence is often recognized at very low frequencies, and it is thought to reflect muscle activation co-modulation (Mochizuki et al., 2006). Previous research showed a higher coherence between lower leg muscles within the same leg with the strongest coherence at lower frequencies (Boonstra et al., 2015). Similar results were found in the present study. There was a strong coherence between the muscles on the same leg and it was stronger for the right leg. This might be because all participants involved in EMG data collection were right-foot dominant and the tandem position reading was taken with the dominant foot (right foot) placed behind the non-dominant foot. In the future, this hypothesis could be examined further by taking the participants who are left foot dominant as well and collecting the tandem position data in two ways, right foot behind the left foot and left behind right.

This is the first study to use a time-frequency coherence analysis to examine the EMG signals of different muscle pairs. Similar results were obtained using this technique for the coherence between muscle pairs. Strong coherence was observed between muscle pairs from the same leg and there was a low coherence between muscle pairs from different legs. Danna-Dos-Santos et al. (2015) has shown intermuscular coherence to be substantial in muscle pairs produced exclusively by posterior or anterior muscles; mixed muscle pairs did not exhibit significant coherence. In our study, as shown in the coherence spectrogram for one representative participant (Figures 9, 10), there appears to be strong coherence between RMG and RS. Coherence appears to increase with the more unstable standing balance position, i.e., tandem stance.

## 5. Limitations

When using surface electrodes for muscles in close proximity there is always the possibility of cross-talk among adjacent muscles. However, our cross-correlation analysis indicated that this phenomenon was negligible. The use of wavelets and MSC is limited to the analysis of linear signals. MSC is appropriate for signals that are non-stationary, but it is a measure of the linear phase relationship between two time series. Since it has been shown that EMG signals are non-linear the use of linear methods of analysis may introduce errors. Surrogate data analysis methods have shown that with movements and fatigue EMG signals are not stochastic but contain deterministic non-linear components (Lei and Meng, 2012). The participants volunteering for the data collection were all right-foot dominant and the tandem position data collection was completed with only the right, dominant on the rear force plate. It would be more useful to support the result of the study if the data were also collected from individuals who are left-foot dominant. Additionally, two tandem positions need to be considered – dominant foot forward, and dominant foot back. We did not randomize trials. The data were collected in serial order; FTEO to FTEC to TanEO to TanEC. In this order of standing balance task, the participants were asked to go from a least difficult stable posture to a complex stable posture. The balance standing task order may not have a large impact on the result of the analysis but randomizing the trials and collecting the data by starting from the more complex standing position may be useful. Also, in the future, cortico-muscular coherence can be estimated under these different postural stability conditions. The use of corticomuscular coherence in a time-frequency domain has been suggested as a means of detecting movement intent (Severini et al., 2012).

## 6. Conclusion

Magnitude-squared-coherence and time-frequency coherence were observed between different muscle pairs from the lower leg on both left and right sides under different stability conditions in different neural frequency bands. Coherence between muscle pairs of the same leg was found to be higher (the highest coherence between the MG and S muscle). Using wavelets, higher coherence and coherence distribution were observed in the low frequency bands during the more unstable standing balance position. Results from this study might be used as a reference to examine the neural functioning of athletes with concussion by studying neuromuscular relation to muscular activation. In the future, this study may help to provide a benchmark to compare the neural function of athletes who have suffered from concussive damage to our normal, healthy control participants.

## Data availability statement

The raw data supporting the conclusions of this article will be made available by the authors, without undue reservation.

## Ethics statement

The studies involving human participants were reviewed and approved by the Institutional Review Board at Grand Valley State University. The patients/participants provided their written informed consent to participate in this study.

## Author contributions

AO collected the data, wrote the code for data analysis, and compiled the results. GA and SR helped with statistical analysis, result interpretation, and the manuscript editing. All authors contributed to the article and approved the submitted version.

## Footnotes

1
https://www.vicon.com/software/models-and-scripts/
